# Cancer immunometabolism: advent, challenges, and perspective

**DOI:** 10.1186/s12943-024-01981-5

**Published:** 2024-04-05

**Authors:** Qin Dang, Borui Li, Bing Jin, Zeng Ye, Xin Lou, Ting Wang, Yan Wang, Xuan Pan, Qiangsheng Hu, Zheng Li, Shunrong Ji, Chenjie Zhou, Xianjun Yu, Yi Qin, Xiaowu Xu

**Affiliations:** 1 Department of Pancreatic Surgery, Fudan University Shanghai Cancer Center, Shanghai, China; 2 Department of Oncology, Shanghai Medical College, Fudan University, Shanghai, China; 3 Shanghai Pancreatic Cancer Institute, Shanghai, China; 4 Pancreatic Cancer Institute, Fudan University, Shanghai, China; 5 School of Clinical Medicine, Zhengzhou University, Zhengzhou, China; 6 Department of Hepatobiliary Surgery, Yijishan Hospital, The First Affiliated Hospital of Wannan Medical College, Wuhu, China; 7 Department of Thoracic Surgery, Shanghai Pulmonary Hospital, Tongji University, Shanghai, China

**Keywords:** Immunometabolism, Metabolic reprogramming, Immunity, Metabolic adaptation, Cancer-immunity cycle, Cancer-immunometabolism subcycle

## Abstract

For decades, great strides have been made in the field of immunometabolism. A plethora of evidence ranging from basic mechanisms to clinical transformation has gradually embarked on immunometabolism to the center stage of innate and adaptive immunomodulation. Given this, we focus on changes in immunometabolism, a converging series of biochemical events that alters immune cell function, propose the immune roles played by diversified metabolic derivatives and enzymes, emphasize the key metabolism-related checkpoints in distinct immune cell types, and discuss the ongoing and upcoming realities of clinical treatment. It is expected that future research will reduce the current limitations of immunotherapy and provide a positive hand in immune responses to exert a broader therapeutic role.

## Highlights

1. Attempting to delineate the complex and multidimensional interplays between metabolites (or metabolic enzymes) and predominant immune cell populations.

2. Metabolic checkpoints of immune cells are described and the contribution of these metabolic targets to determine the metabolic adaptations of distinct immune cells in specific tissue environments is emphasized.

3. Proposed cancer-immunometabolism subcycle, enriching the theoretical foundation of the cancer-immunology field.

4. Metabolism-induced disturbances in the acid-base balance of the tumor microenvironment have non-redundant effects on immunotherapy.

5. Pending challenges and clinical concerns in metabolic insights were addressed.

## Introduction

As a pioneer in the quantitative study of cancer cell metabolism as well as photosynthesis and respiration, Otto Warburg and colleagues first unraveled the mystery of cancer’s ability to rapidly consume large amounts of glucose independent of oxygen for its growth and proliferation in the 1920s, a phenomenon also known as the Warburg effect [1, 2]. Indeed, various of solid tumors exhibit the Warburg effect while preserving mitochondrial respiration, which is an inefficient way to generate adenosine 5’-triphosphate (ATP), compared to oxidative phosphorylation (OXPHOS) [3]. Studies reported that the primary function of the Warburg effect may be to maintain high levels of glycolytic products, or even to enable “metabolic transformation” to support active anabolic reactions within the cell [4, 5]. Similarly, a consequence of oxidative metabolism is the production of reactive oxygen species (ROS), which could support tumorigenesis but require tight regulation of redox balance [6]. Of interest, tumors undergo dysregulation of multiple metabolic pathways improve the metabolic flexibility, which subsequently induces altered immune status and tumor progression [3, 7, 8]. For instance, certain metabolic processes are aberrantly enabled in cancer cells, including glutamate transport, rapid glutamine uptake, and fatty acid oxidation (FAO), which involve metabolites that act as immune mediators, resulting in reduced immunogenicity of cancer cells, immune escape, as well as state of localized immunosuppression in the tumor microenvironment and immunotherapy resistance [9–12]. However, the paucity of successful clinical data on metabolism-related therapies in cancer patients continues to attract researchers to initiate more in-depth studies.

**Fig. 1:**
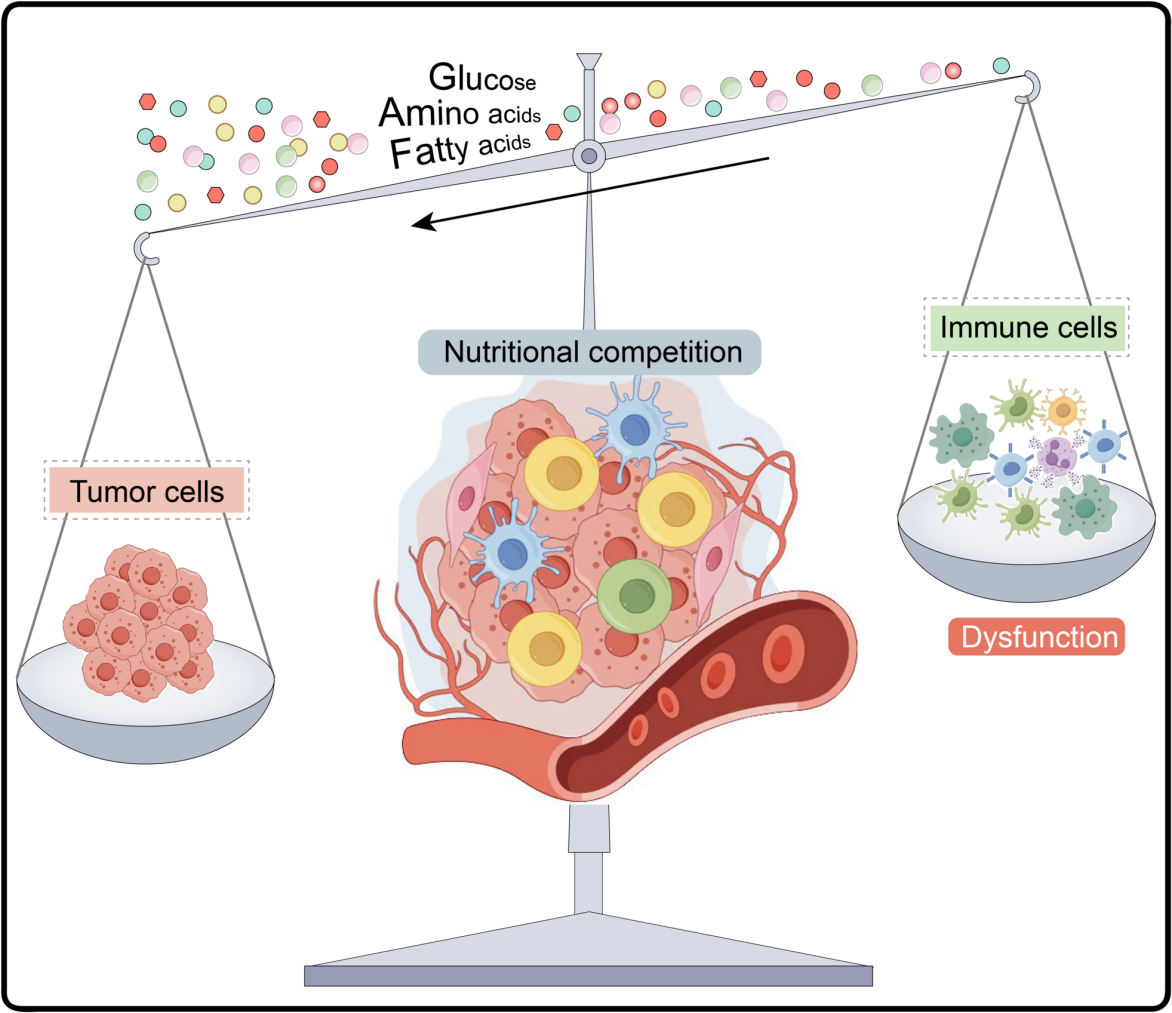
Metabolic competition between tumor cells and immune cells. The availability of nutrients for metabolic processes is fundamental for cell survival, along with tumor cells and immune cells are no exception. Competitive uptake of nutrients by tumor cells in the tumor microenvironment may occur at all stages of immune cell life. Metabolite paucity tilts the energy balance in favour of the tumor cells (the negative direction), which in turn leads to further dysfunction of immune cells (such as naïve T cells, B cells, natural killer cells, macrophages, neutrophils, and dendritic cells, etc.)

The perception of metabolism by immune cells is closely linked to their fate decisions [13–16]. Nonetheless, it is inevitable that cancer cells are inherently competitive with immune cells in their demand for essential nutrients [17, 18]. The nutrient competition is depicted in Fig. 1. Chang and colleagues demonstrated metabolic competition between tumor cells and T cells in a mouse sarcoma model, which contributes to T cell dysfunction and tumor progression [19]. Hypoxia, one of the key drivers of tumor heterogeneity, mediates both metabolic reprogramming and immune escape [20]. Cholesterol metabolism produces important membrane components as well as metabolic derivatives with diverse biological functions [21]. Preclinical and clinical studies have shown that manipulation of cholesterol metabolism suppresses tumor growth and remodels the immune landscape [22, 23]. Specifically, the metabolic demands of immune cells largely affect the success of immunotherapy, which might be one of the principal reasons why many cancers remain resistant to immunotherapy and the long-term prognosis of patients cannot be guaranteed [17, 24]. Ultimately, if immunotherapy could be used early in the disease or as a link in combination therapy, the initiation of immune responses and transformation of the immunophenotype might be less restricted and perhaps more malleable.

With the proposed cross-cutting field of immunometabolism, the immune system underlying the metabolic landscape is being redefined by oncologists from multiple perspectives [25–28]. This review attempts to delineate the complex and multidimensional crosstalk between metabolites (or metabolic enzymes) and predominant immune cell populations, and highlights the contributions made by metabolic targets to the metabolic adaptations of immune cells in specific environments. Accordingly, clinical oncology treatment has progressed based on attempts to combine nutritional therapies with immunotherapy, yet there are still open questions.

### Metabolic reprogramming for immune regulation

Accumulating evidence has led oncologists and immunologists to appreciate that metabolites and enzymes are important regulators of the immune system, which involved in energy circuits and signaling cascades [3, 29, 30]. Therefore, metabolic reprogramming caused by abnormal metabolites or metabolic enzymes produces a profound effect on the immune response.

### Metabolites act as immune mediators

Metabolites have functions in the immune system independent of their traditional roles as biosynthesis and energy supply [31]. However, most studies to date have focused on the regulation of metabolic pathways during immune responses [32]. Notably, the discovery of metabolites and intermediates as novel signaling molecules is thought to produce a profound effect on immune regulation [33, 34].

#### Glucose

**Fig. 2:**
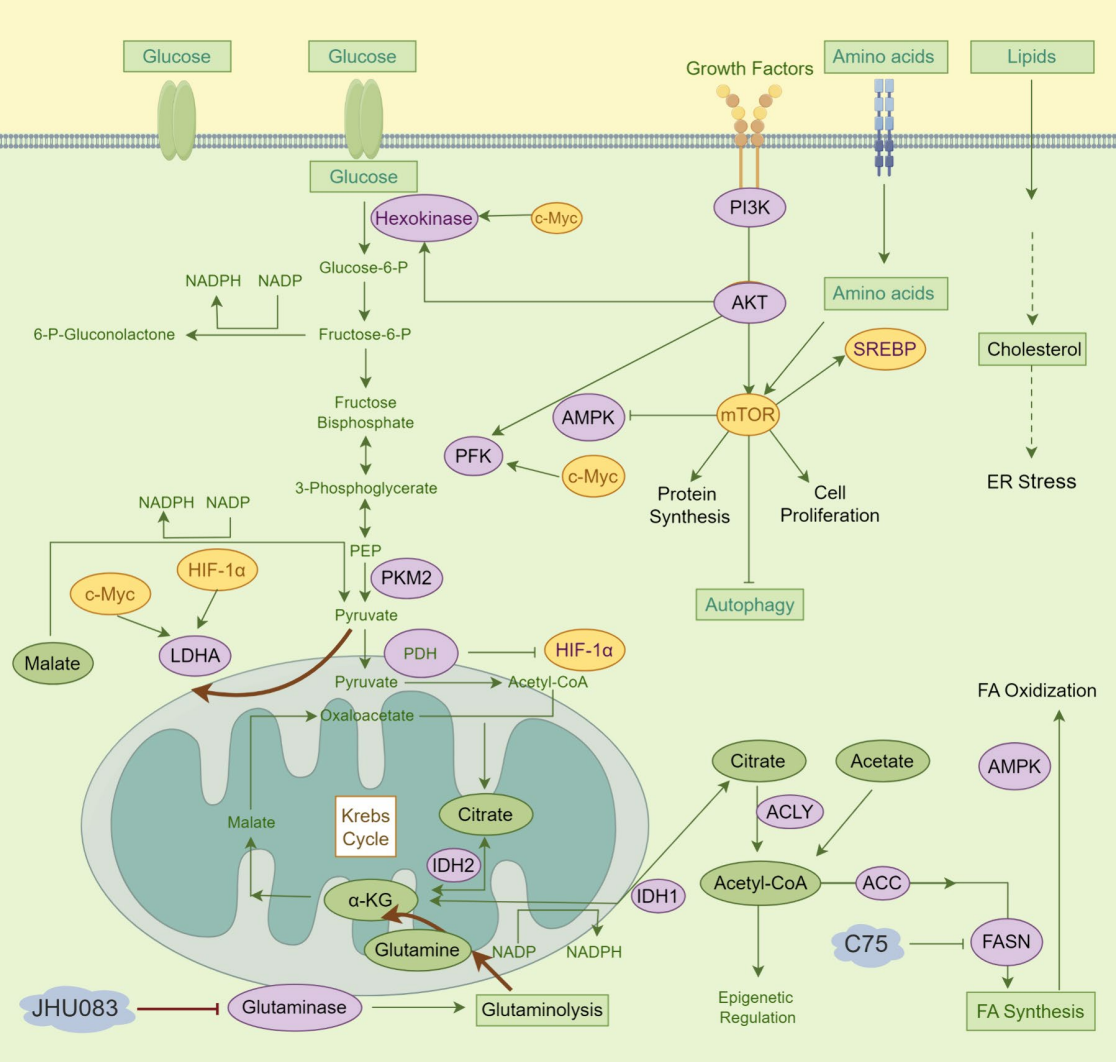
Immune-related intracellular energy metabolism and substance synthesis. The diagram shows the metabolic activities and synthetic reactions that occur in the cell under enzymatic reactions and correlate with or might cause immune changes (enzymes are labeled in purple, nutrients or metabolites are labeled in green, molecules or targets are labeled in yellow, and inhibitors are labeled in grey). For example, lipid uptake from the TME leads to elevated intracellular cholesterol concentrations, which in turn triggers ER stress (inducing CD8^+^ T cell dysfunction). The PI3K/AKT pathway, which is activated by growth factor signals, stimulates the mTOR family molecules, which in turn elicits vital activities such as protein synthesis, cell proliferation, and autophagy, and so on. The mTOR family molecules are also regulated by amino acids. FA synthesis is coordinated sequentially by several enzymes involving ACC1 (inhibition of ACC1 reduces TH17 cell differentiation but enhances the formation of memory CD4+ T cells). And C75 inhibits FASN which in turn diminishes FA synthesis. In addition, the induction of FA oxidation was associated with an increase in AMPK activity (AMPK also promotes the generation of memory CD8^+^ T cells). JHU083 inhibited glutaminase-mediated glutaminolysis. TME, tumor microenvironment; SREBP, sterol regulatory element binding protein; ER, endoplasmic reticulum; PI3K, phosphatidylinositol 3-kinase; FA, Fatty acid; ACC1, acetyl-CoA carboxylase 1; PDH, pyruvate dehydrogenase; αKG, α-ketoglutarate; FASN, fatty acid synthase; AMPK, AMP-activated protein kinase

Glucose supply and glycolysis processes play an important role in the development and progression of tumors (Fig. 2) [35]. CD8^+^ T cell proliferation and cytokine production depend on enhanced glucose metabolism [36]. Glucose restriction can activate AMPK-coupled SENP1-Sirt3 signaling in mitochondria and promote T cell development [37]. Animal studies have indicated that diabetic mice exhibit larger breast tumors characterized by altered collagen structure, increased tumor-allowed M2 macrophage infiltration, and early spread metastasis [38]. Competitive glucose metabolism may also be a target to improve the efficacy of bladder cancer immunotherapy [39]. Notably, a recent study has focused on glucose-promoting tumor progression and immunotherapy resistance in a non-classical metabolism-dependent manner, directly in the form of signal transduction molecules [12]. Collectively, these studies not only show that glucose levels play an important role in the energy interaction between tumors and immune cells, but also highlight the role of glucose molecules as signaling molecules for immune regulation from a new perspective.

#### Amino acids

Tumor growth and development depend on the intake of foreign amino acids, which affects the function of immune cells [40, 41]. Therefore, alterations in amino acid metabolism could be used not only as a clinical indicator of cancer progression but also as a therapeutic strategy.

### Leucine (Leu) & Arginine (Arg)

As one of the branched-chain amino acids, leucine (Leu) acts as a nitrogen donor to produce biomolecules such as nucleotides, which are indispensable for the growth of cancer cells [42]. In a clinical study (NTR2121), a nutritional intervention with a high-leucine specific medical food rapidly increased the percentage of EPA and DHA in leukocyte phospholipids and lowered serum levels of the inflammatory mediator PGE2 within one week in cancer patients undergoing radiation therapy [43]. Leu restriction has now been shown to limit the response of premalignant B cells. It is now well established that limiting leucine then limits the response of pro-cancer B cells [44, 45]. Arginine (Arg) metabolism affects not only malignant cells but also the behavior of surrounding immune cells [46, 47]. Inhibition of Arg by CB-1158 blocked myelocyte-mediated immunosuppression in the tumor microenvironment (TME) [48]. Miret et al.’s data suggests Arg is an immunomodulatory target in KRAS^G12D^ genetically engineered mouse models, and inhibition of arginase attenuated tumor growth [49]. Therefore, it is promising to develop therapeutic strategies targeting immunomodulatory pathways controlled by Leu and/or Arg degradation.

### Glutamate (Glu) & glutamine (Gln)

Glutamate (Glu) is a major excitatory neurotransmitter in the central nervous system (CNS) and also plays a critical function in tissue and cellular metabolism through the tricarboxylic acid cycle [50, 51]. Long et al. found that dysregulated Glu transport enhances T regulatory cell (T_reg_ cell) proliferation, activation, and immunosuppressive functions, as well as promotes resistance to VEGF blockade of glioblastomas in vitro [9]. The use of a glutaminase antagonist, JHU083, effectively inhibited tumor growth in a variety of solid tumor models and significantly improved mouse survival [52]. Furthermore, activation of naïve T cells is associated with rapid Gln uptake [10]. Selective inhibition of Gln metabolism in tumor cells increases anti-tumor T lymphocyte activity in triple-negative breast cancer (TNBC) patients [53]. Thus, Glu and Gln metabolism are reprogrammed during tumorigenesis and are considered a promising target for cancer therapy.

### Tryptophan (Trp) & Asparagine (Asn)

Substantial evidence suggests that tryptophan (Trp) and asparagine (Asn) metabolism are physiologically and pathologically involved in the progression and treatment of a wide range of diseases, including cancer [54–56]. Qin et al. found that IDO inhibitors could mediate tryptophanyl-tRNA synthetase (WARS) overexpression *via* accumulating Trp, which accelerates TRIP12 tryptophanylation and reduces surface PD-1 of mouse CD8^+^ T cells [57]. Thus, supplementation with exogenous Trp or use of IDO inhibitors to impede Trp catabolism may be beneficial for PD-1 blockade therapy. Likewise, it has been demonstrated that the Trp metabolizing enzyme tryptophan 2,3-dioxygenase (TDO) inhibits the anti-tumor activity of CD8 T cells in TNBC [58]. Additionally, Trp metabolism mediates impaired differentiation of myeloid cells infiltrated by IDH mutant gliomas, resulting in an immature immune phenotype [59]. Recent evidence suggests that Trp metabolites released by Lactobacillus tumefaciens locally promote interferon-gamma (IFN-γ)-producing CD8 T cells, thereby enhancing immune checkpoint inhibitor efficacy [60]. The intimate link between the kynurenine (Kyn) pathway of tryptophan metabolism and T cell function has been widely reported to date. As proof, IDO inhibitors enhance CD8^+^ T cell effects by accumulating tryptophan and or inhibiting Kyn production [57, 61]. A dietary model constructed by Siska et al. demonstrated that a high concentration (1 mM) of D-kyn could inhibit T cell proliferation *via* apoptosis manner [62]. Kyn derivatives 3-hydroxyanthranilic acid inhibits pro-inflammatory factors in several cell subsets including resident macrophages, proliferating macrophages, and plasmacytoid dendritic cells (pDCs) [63]. Nevertheless, in a study that enrolled 891 non-small cell lung cancer (NSCLC) samples, Bessede et al. noted that combined anti-PD-1/PD-L1 targeting of IDO1 might only be beneficial in patients with inflammatory tumors, and that the IDO1 pathway in NSCLC is driven by the immune system rather than tumor cells [64]. Correspondingly, the SRC family protein tyrosine kinase LCK is phosphorylated at tyrosine 394 and 505 upon binding to Asn, which subsequently increases T cell activation and anti-tumor effects [65]. Emerging evidence reveals that Asn restriction allows for increased metabolic capacity and anti-tumor function in CD8^+^ T cells in an NRF2-dependent manner of enhanced stress response [66]. To sum up, studies on amino acid metabolism and immunomodulation and immunotherapy or combination therapy based on amino acid metabolism still need to be further explored.

#### Lipids

**Fig. 3:**
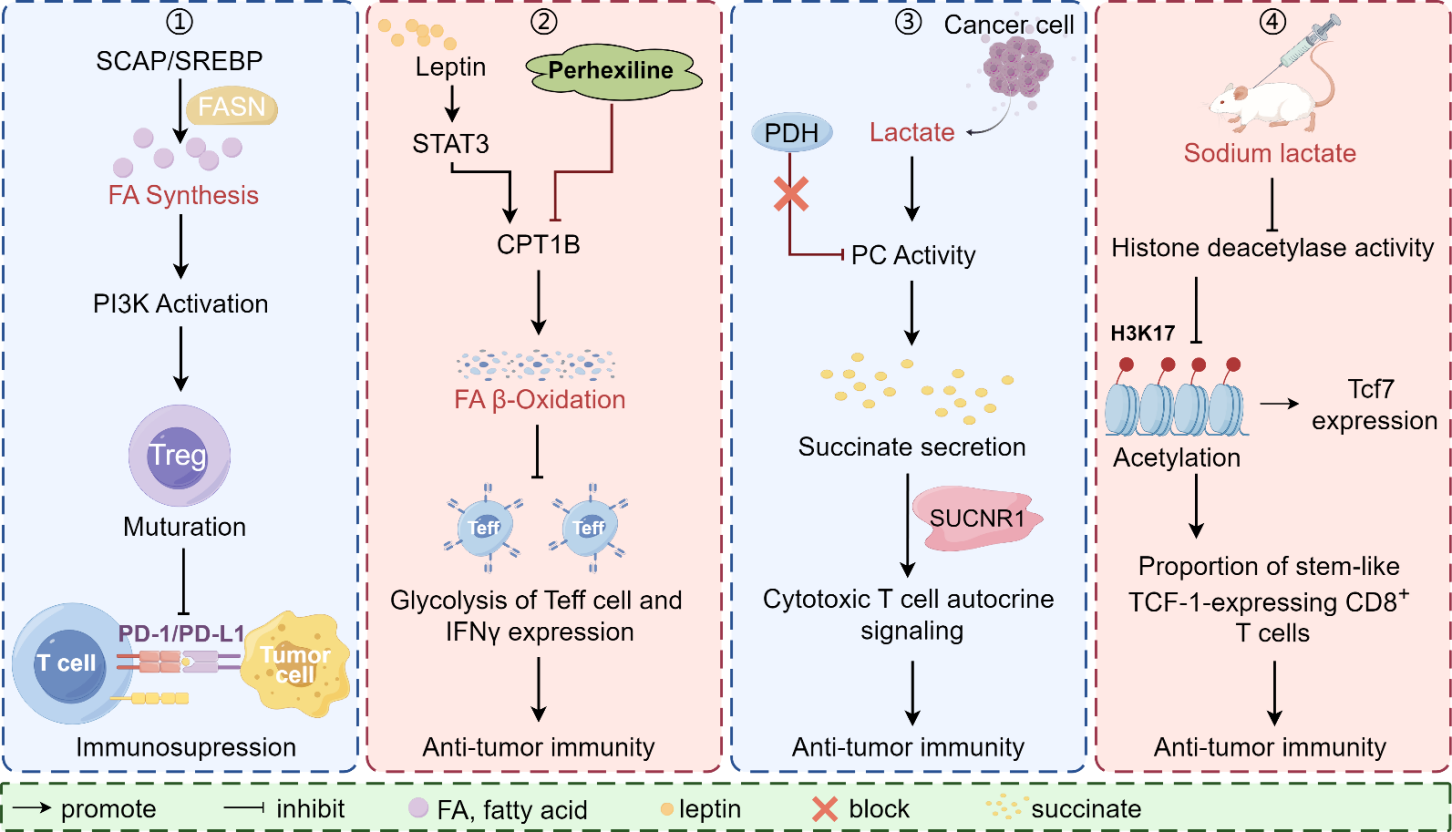
Double-edged swords in cancer immunometabolism. Implications of FA synthesis (1) and catabolism (2) on tumor progression. 1) Upregulation of SREBP activity in T_reg_ cells synergizes with FASN to promote FA synthesis, which in turn activates the PI3K pathway and facilitates the maturation of T_reg_ cells. Specific deletion of SCAP (an essential factor for SREBP activity) by T_reg_ cells enhances anti-PD-1 immunotherapy; 2) Leptin downregulates CD8^+^ T cell effector function through activation of STAT3-FAO and inhibition of glycolysis. Ablating T cell STAT3 or treatment with perhexiline (FTO inhibitor) in obese mice spontaneously developing breast tumor reduces FAO, increases glycolysis and CD8^+ ^T effector cell functions, leading to inhibition of breast tumor development. Additionally, the effects of lactate on cancer and immune cells in TME can be complex and difficult to decipher, which is further confounded by acid protons (byproducts of glycolysis). 3) Tumor-derived lactate is an inhibitor of CD8^+^ T cell cytotoxicity. Cytotoxic T cells shunt succinate out of the TCA circulation to promote autocrine signalling *via* the succinate receptor (SUCNR1). Moreover, cytotoxic T cells rely on PC to replenish succinate. Lactate decreases PC activity, and similarly, inhibition of PDH restores PC activity, succinate secretion, and SUCNR1 activation; 4) Lactate increases CD8^+^ T cell stemness and enhances anti-tumor immunity. Subcutaneous injection of lactate in mice transplanted with MC38 tumors leads to CD8^+^ T cell-dependent tumor growth inhibition. Mechanistically, lactate inhibits histone deacetylase activity, leading to increased acetylation of the Tcf7 super-enhancer site, H3K27, which results in increased Tcf7 gene expression. FA, fatty acid; SREBP, sterol regulatory element binding protein; FASN, fatty acid synthase; PI3K, phosphatidylinositol 3-kinase; FAO, fatty acid oxidation; TME, tumor microenvironment; PC, pyruvate carboxylase; PDH, pyruvate dehydrogenase

Lipid-rich lung-resident mesenchymal cells (MCs) are known to promote lung metastasis of breast cancer. Lipid-loaded MCs transport lipids to tumor cells and natural killer (NK) cells *via* exosome-like vesicles, leading to enhanced tumor cell survival and proliferation as well as NK cell dysfunction [67]. Accordingly, lipid droplets are intracellular lipid reservoirs that are utilized by effector memory CD4^+^ T cells in nutrient-deficient environments [68]. Cholesterol metabolism plays a crucial role in regulating anti-tumor immune responses by acting on various immune cells involved in innate and adaptive immune responses [69, 70]. In addition, caloric restriction decreases total cholesterol and triglyceride levels, stimulates cancer immune surveillance, and reduces the migration of immunosuppressive regulatory T cells to tumors [71]. Cholesterol in TME induces dysfunctional CD8^+^ T cells by triggering endoplasmic reticulum (ER) stress, manifested by certain co-inhibitory molecule expression and impaired effector function [72]. In addition to serving as a fuel source for energy production, fatty acid (FA) primarily serves as structural components of membrane matrices and important secondary messengers (Fig. 2) [73, 74]. FA synthesis is coordinated by several enzymes involving acetyl-CoA carboxylase 1 (ACC1), and inhibition of ACC1 decreases TH17 cell differentiation but enhances the formation of memory CD4^+^ T cells [75–77]. Induction of FAO is associated with an elevation of AMP-activated protein kinase (AMPK) activity, while AMPK promotes the generation of central memory CD8^+^ T cells [78, 79]. Grajchen et al. found that enzyme-catalyzed desaturation of FAs is an important determinant of T_reg_ differentiation and autoimmunity [80]. FAs play a double-edged role in the immunomodulation of the body (Fig. 3) [11, 81]. In addition, studies on lipid and lipoprotein transport pathways have provided options for improving prelipidic routes of administration for oral administration and therapy, with the promise of involvement in immunotherapy through the lymphatic system [82, 83]. Thus, the importance of altered cholesterol and FA metabolism in cancer should receive new attention.

### Other alternative metabolites

#### Citrate

Citrate produces a profound effect on the immune and inflammatory responses engaged in both primary and adaptive immune cells and is also thought to play a crucial role in cancer metabolism [15, 84, 85]. Studies have shown that citrate could induce macrophages to rapidly secrete pro-inflammatory cytokines, which in turn facilitates the destruction of cancer stem cells (CSCs) [86]. Inhibition of citric acid carriers could cause peripheral macrophage inactivation and reduce cerebral thrombosis [84]. In fact, carcinogenic signaling pathways, such as HIF-1α and RAS/PI3K/AKT, may cause resistance by enhancing the aerobic glycolysis of cancer cells, known as the “Warburg effect” [86–88]. However, most drugs are weakly alkaline molecules, and this metabolism that promotes the development and aggressiveness of cancer cells can also induce increased extracellular acidity to weaken the penetration of compounds into cancer cells and even lead to the occurrence of multi-drug resistance events. Interestingly, citrate-rich organs, such as the liver, brain, and bone, are also common sites for metastasis of various malignant tumors, and it is possible that high citrate forms a good metastatic niche for the growth of secondary tumors and improves the survival rate of colonized cancer cells [89, 90]. Therefore, it might be possible to enhance the local infiltration of tumor chemotherapy drugs by reducing extracellular acidity strategies to achieve effective therapeutic concentrations and improve prognosis in clinical or preclinical trials.

### Other mitochondria and TCA cycle metabolites

Most of the intermediates in the TCA cycle are important raw materials for the synthesis of the three nutrients (glucoses, lipids, and proteins), so the TCA cycle is also considered to be the hub of the metabolic connection of the three nutrients [91, 92]. The TCA cycle and oxidative phosphorylation process in mitochondria could produce a variety of metabolites and energy substances including ATP, NADH, α-ketoglutaric acid, etc., and further cross-link with immune cells (Fig. 2) [93–95]. For instance, as a by-product of dehydrogenase reaction in the electron transport chain, NADH is involved in regulating the energy metabolism of immune cells [94]. And α-ketoglutarate (αKG) is an intermediate product involved in energy metabolism and synthesis of T cell proliferation and inflammation [95, 96].

### PGE2 and lactate

Conventional type 1 dendritic cell (cDC1) is an important anti-tumor immune cell, which can present tumor antigens and secrete IL-12 and other cytokines that promote T cell activation and effector function. Studies have confirmed that PGE2 can directly inhibit the survival of NK cells and the production of chemokines, and reduce the reactivity of cDC1 to chemokines, thereby blocking the recruitment of cDC1 [97]. Using a variety of mouse tumor models, researchers have found that the endogenous cyclooxygenase-2 / prostaglandin E2 (COX-2/PGE2) pathway in tumor cells inhibits NK cell infiltration and IFN-γ production, thereby promoting tumor evasion from immune surveillance [98]. Watson et al. reported that lactate treatment prevented the damaging effects on the function and stability of T_reg_ cells under high glucose conditions [99]. In addition, lactic acid accumulation in the tissue microenvironment limits the function of immune cells, yet activated immune cells need lactic acid to perform their functions [100–102]. Briefly, lactate reduces pyruvate carboxylase (PC) activity, succinate secretion and SUCNR1 activation, which in turn inhibits autocrine signaling in cytotoxic T cells [102]. Therefore, lactate produces a double-edged effect on the immune process, suggesting a complex link between tumor immunity and metabolite regulation (Fig. 3) [100, 102].

### Metabolic enzymes act as immune mediators

Inevitably, enzymatic reactions catalyzed by metabolic enzymes play a non-negligible role in the synthesis or catabolism of metabolites, especially in pathological states [103]. Changes resulting from metabolic enzyme abnormalities may be efficient and specific. Therefore, unlike nutritional therapies related to metabolite deficiencies, specific blocking agents are also used for treatments or study against metabolic enzyme abnormalities.

#### Glycolytic rate-limiting enzyme

Hexokinase 2(HK2) is one of the key protein kinases in the glycolysis pathway, which is mainly located in the mitochondrial outer membrane and could regulate the permeability of mitochondrial membrane [104, 105]. HK2 is usually induced to catalyze glucose metabolism in cancer cells and is highly expressed in various tumors, including prostate cancer, liver cancer, gastric cancer (GC), glioblastoma, and breast cancer [105–110]. As a sensor in the first step of catalytic gluconeogenesis, HK2 could exert regulatory effects independently of downstream glycolysis reactions. To illustrate, glucose is involved in inducing upregulation of programmed cell death ligand 1 (PD-L1) expression in glioblastoma *via* HK2 in a dose-dependent manner, and that this induction process is independent of oxygen availability [108]. HK2 also acts as an A-kinase anchoring protein (AKAP) to increase the stability of GSK3 targets, mediating SNAIL glycosylation to promote epithelial-mesenchymal transformation (EMT) in mouse models of BC metastasis, which is independent of glucokinase activity of HK2 [109]. Identification based on the HK subtype showed that most of the non-tumor tissues expressed only HK1, while most of the tumor tissues expressed both HK1 and HK2 [110]. Therefore, the investigation of HK2 and its regulation of the tumor immune microenvironment may remain unclarified.

Another supervisor of the glycolytic pathway, phosphofructokinase 1(PFK1) could catalyze the irreversible conversion of fructose-6 phosphate (F6P) and ATP to fructose-1, 6-diphosphate (F1,6BP) and ADP [111]. PFK1 accelerates glycolysis and the formation of a local acidic microenvironment in tumors [112]. Additionally, extracellular acidity promotes invasion, immunosuppression, and therapeutic resistance [113–115]. Activation of phosphofructokinase-1 liver type (PFKL) and inhibition of the pentose phosphate pathway suppresses NOX2-dependent oxidative burst in neutrophils [116]. Transforming growth factor-β (TGF-β) increased PFKL expression and activity during macrophage activation, promoting glycolysis but inhibiting pro-inflammatory cytokine production [117]. Importantly, numerous in vitro studies confirm that administration of high concentrations of citrate, a potent physiological inhibitor of PFK1 and PFK2, reduces ATP production, induces apoptosis, and sensitizes cells to cisplatin treatment [86, 118, 119]. Therefore, an in-depth understanding of the role of PFK1 in the maintenance of immune homeostasis and disease progression is essential to help probe the overall regulation and mutual collaboration between cellular metabolic activities and immune regulation.

#### Fatty acid synthase (FASN)

Fatty acid synthase (FASN) meets the energy requirements of tumor cells during growth and proliferation by de novo synthesizing of FAs, and promotes various malignant phenotypes in tumors [120]. FASN also connects to cellular metabolism and tumor immunomodulation [121]. As proof, a pan-cancer analysis performed by Zhang et al. showed that the expression level of FASN was significantly negatively correlated with the immune infiltration in 35 tumors and immunotherapeutic targets (including PD-1, PD-L1 and CTLA-4, etc.) in 15 tumors [122]. Synthesis of new FAs mediated by FASN contributes to the functional maturation of T_reg_ cells, and loss of FASN in T_reg_ cells inhibits tumor growth [11]. In addition to reducing fat accumulation in hepatocytes, inhibition of FASN directly suppresses immune cells and stellate cells [123]. Phosphatidylinositol 3-kinase alpha (PI3Kα)-specific inhibitor CYH33 promotes the FA metabolism in TME and ultimately enhances the immune response in combination with the FASN inhibitor C75 [124]. This CYH33-driven process involves preferential M1 polarization of macrophages and increased activity of CD8^+^ T cells. The above suggests that targeting FASN may improve immunotherapy by altering the local immune microenvironment of tumors.

#### Lysosomal acid lipase (LAL) & ATP citrate lyase (Acly)

Lysosomal acid lipase (LAL), encoded by the lipase A gene, is the only lysosomal enzyme responsible for catalyzing the hydrolysis of cholesteryl esters and triglycerides at acidic pH [125, 126]. Huang et al. reported that LAL is involved in and ultimately determines M2 activation in macrophages [127]. LAL deficiency was reported to cause systemic expansion and infiltration of myeloid-derived suppressor cells (MDSCs) in multiple organs [128]. In the blood of LAL-deficient (Lal-/-) mice, an increase in CD11c^+^ cells were observed to be accompanied by an upregulation of PD-L1 expression, which may also cause value-added tumors in the bone marrow of mice [125, 129]. Another study observed suppressed immune rejection and allowed human lung cancer cell growth in Lal-/- mice [130]. Explicitly, O’Sullivan et al. revealed that memory T cells rely on cell-intrinsically expressed LAL to mobilize FA to support FAO and memory T cell development [131].

ATP citrate lyase (Acly), which converts citrate to acetyl-CoA in the cytoplasm, is one of the major enzymes catalyzing the formation of cytosolic acetyl-CoA [90, 132]. Acetyl-CoA synthesized by Acly plays an essential role in mitochondrial metabolic processes such as acetylation and lipid synthesis of various proteins [133–135]. Pharmacological analyses report that intracellular acetyl-CoA enhances the therapeutic effect of CD8^+^ T cells [85]. Toll-like receptor signaling re-mediates macrophage metabolism and promotes histone acetylation *via* Acly [136]. Acetyl-CoA production was dependent on the glucose transporters GLUT3 and Acly is a promising metabolic checkpoint for alleviating Th17 cell-mediated disease [137]. Furthermore, Acly-dependent histone acetylation promotes hematopoietic stem cell differentiation to CD48^+^ progenitors [90]. Notably, Acly was actively degraded during the differentiation of in vitro-derived T_reg_ cells, leading to downregulation of FA synthesis to support T_reg_ cell generation [138]. The above results highlight the global impact of LAL or Acly deficiency on metabolic homeostasis and immune cell function, and play a profound role in the metabolic regulation of cellular immunity.

#### Isocitrate dehydrogenase (IDH)

Isocitrate dehydrogenase (IDH) 1 and 2 (IDH1 and IDH2) are the most frequently mutated metabolic genes in human cancers [139–141]. IDH1 could play a key role in lipogenesis and maintenance of redox homeostasis in mammalian hepatocytes [142]. It has been shown that gain-of-function mutations in IDH in human cancers lead to the production of d-2-hydroxyglutarate (d-2HG), a metabolite that promotes tumorigenesis through epigenetic alterations and can alter T-cell metabolism and impair CD8^+^ T-cell function [143]. Defective metabolism of IDH was identified in M1 macrophages [144]. In addition, enzymatic properties of mutant IDH1 inhibited IFNγ-TET2 signaling and promoted immune escape and tumor viability in cholangiocarcinoma [145]. IDH mutations are highly correlated with the degree of intra-tumor heterogeneity, and IDH mutations produce a paracrine metabolite, (R)-2-hydroxyglutarate, which can be involved in shaping the tumor immune microenvironment [146, 147]. A high proportion of tumor-associated macrophage subpopulations mediating antigen presentation was found in IDH-mutated grade 4 astrocytomas [148]. In a mouse glioma model, treatment with mutant IDH1 reduced levels of the chemokine CXCL10 and inhibited T cell aggregation at the tumor site [149]. In conclusion, information about the association between IDH and immune cell regulation remains to be explored.

#### Hyaluronidase (HAase)

Hyaluronidase (HAase) on the cell surface hydrolyzes hyaluronic acid (HA) improves fluid permeability in tissues, and is a potent modulator of ER stress resistance [150]. It is generally accepted that HAase significantly improves the efficiency of percutaneous drug delivery and assists local anesthesia in reducing operative pain [151, 152]. HAase plays a pro-cancer role in a variety of cancers [153]. To illustrate, HAase accumulates at sites of inflammation in the body and accelerates the degradation of HA, which is present in high levels in the skin, thereby modulating tumor cell invasion and angiogenesis and protecting against immune cell attack [154]. In addition, HA stimulates the expression of various immune cells at the site of injury [155, 156]. Blair et al. reported that degradation of HA in combination with anti-PD-1 antibody and focal adhesion kinase inhibitor reduced granulocytes [157]. Of interest, Liu et al. developed a nanosystem that evidently increased HAase activity, which synergized with light irradiation could reduce HIF-1α expression and infiltration of immunosuppressive cells in breast cancer model [158]. In terms of clinical translation, HAase-mediated cascade degradation of the stromal barrier and immune cell penetration by microneedles enable efficient anti-tumor therapies [159]. Therefore, HAase is considered a potential target that can modulate immune cell metabolism and mediate immunotherapy that cannot be ignored.

### Metabolic checkpoints: trigger targets for changing metabolic manners in distinct immune cell populations

Briefly, the regulatory role of metabolites (or metabolic enzymes) in immune responses was addressed above from the metabolic perspective. Therefore, this section focuses on the molecular targets and signaling pathways that alter the metabolic patterns of these immune cell populations. Referring to the immunologists’ nomenclature of immune checkpoints (targets that regulate autoimmune responses), we roughly named these targets that can change the metabolic pattern of immune cells as “metabolic checkpoints”. We emphasize the impact of metabolic checkpoints on the functional differentiation and fate determination of immune cells, aiming to reveal how metabolic networks mediate the function of specific subtypes of immune cells. Additionally, identifying the metabolic adaptability of different immune cells in specific tissue environments helps us to understand how these cells defend against pathogens and tumors, and how they maintain tissue health at barrier sites [13, 160]. Ultimately, we summarized multiple immunometabolism axes consisting of metabolites, metabolic checkpoints, and immune cell subpopulations (Table 1).

**Table 1:** Evolving roles alters the immunometabolism of cancer cell survival and growth

**Metabolites**	**Target immune cell**	**Mediators**	**Effects on immunity**	**Outcomes**	**Refer to Refs.**
Glu	T_reg_ cell	mGlutR1	Enhancement of T_reg_ proliferation, activation, and immunosuppression	Pro-cancer	[9]
FA	T_reg_ cell	SREBP and FASN	SREBP combined with FASN promotes FA synthesis, which in turn promotes T_reg_ maturation and drives immunosuppression	Pro-cancer	[11]
			Inhibition of FASN reduces fat accumulation in hepatocytes and directly suppresses immune cells and stellate cells	Anti-cancer	[122]
	CD8^+^ T cell	STAT3	The leptin-STAT3 axis increases oxidation of FAs within CD8^+^ T cells in breast cancer	Pro-cancer	[81]
		CD36	CD36 mediated uptake of FA by CD8^+^ T cells, induced lipid peroxidation and ferroptosis, and led to reduced cytotoxic cytokine production	Pro-cancer	[161]
	B cell	CD37	As essential membrane-localized inhibitor of FA metabolism, CD37 inhibits FATP1 (FA transporter) and subsequently leads to the inhibition of FA metabolism in aggressive B cell lymphomas	Anti-cancer	[162]
isoDCA	DCs and T_reg_ cell	NR1H4; Foxp3	isoDCA inhibits the transcriptional activity of NR1H4 in DCs and attenuate the immunostimulatory properties of DCs, which in turn induces Foxp3 expression and T_reg_ cell generation	Anti-cancer	[16]
Cholesterol	Neutrophil and CD8^+^ T cell	CXCR2	Depletion of toxic γδ-T cells promotes breast cancer metastasis	Pro-cancer	[23]
	Tc9 cell	LXR	Cholesterol or its derivative oxysterols inhibited IL-9 expression by activating LXR Sumoylation-NF-κB signaling	Pro-cancer	[163]
	Neutrophil	CXCR2	Recruiting neutrophils to achieve a state of local TME inhibition	Pro-cancer	[164]
		HIF1α	Promoting angiogenesis in pancreatic neuroendocrine tumors	Pro-cancer	[165]
Leu	B cell	LARS2	LARS2 knockdown or leucine blockade reduces LARS2-expressing B-cell subpopulations, which in turn inhibits immune escape in colorectal cancer	Anti-cancer	[44]
Arg	CD8^+^ T cell	ARG1	Extracellular ARG1could interact with cathepsin S and enhanced enzymatic activity at physiological pH, and NET-associated hARG1 suppresses T lymphocytes	Pro-cancer	[166]
PGE2	NK cell and cDC1	may be XCL1 and CCL5	Direct inhibition of NK cell survival and chemokine production that followed by downregulation of chemokine expression in cDC1	Pro-cancer	[96]
Lactate	T_reg_ cell	MCT1	Upregulation of lactate metabolic pathways to maintain immune response	Anti-cancer	[98]
	Cytotoxic T cell	SUCNR1	Lactate reduces PC activity, succinate secretion and SUCNR1 activation, which inhibits autocrine signaling in cytotoxic T cells	Pro-cancer	[102]
αKG	Macrophage	Jmjd3; NF-κB	Coordinates M2 type activation of macrophages; Impairment of the pro-inflammatory response of M1 macrophages	Pro-cancer	[95]
Glycerol	Memory CD8^+^ T cells	IL-7	IL-7 signaling induces glycerol uptake to promote triacylglycerol synthesis and FAO, thus supporting the longevity of memory CD8^+^ T cells	Anti-cancer	[167]
Trp	CD8^+^ T cell	I3A	*L. reuteri* promotes interferon-gamma-producing CD8^+^ T cells that thereby enhancing immune checkpoint inhibitor therapy	Anti-cancer	[60]
Asn	CD8^+^ T cell	NRF2	Asn restriction enhances the metabolic capacity and anti-tumor function of CD8^+^ T cells	Anti-cancer	[66]

*CXCR2* C-X-C motif chemokine receptor 2, *FA* Fatty acid, *PGE2* Prostaglandin E2, *HIF1α* Hypoxia-inducible factor-1α, *isoDCA* 3β-hydroxydeoxycholic acid, *DCs* Dendritic cells, *TME* Tumor microenvironment, *TANs* Tumor-associated neutrophils, *NK cell* Natural killer cell, *cDC1* Conventional type 1 dendritic cell, *Tc9 cell* IL-9-secreting CD8^+^ T cell, *LXR* Liver X receptor, *Arg* Arginine, *ARG1* Arginase 1, *NETs* Neutrophil extracellular traps, *hARG1* Human arginase 1, *PC* Pyruvate carboxylase, *αKG* α-ketoglutarate, *NF-κB* Nuclear factor-κB, *I3A* Indole-3-aldehyde, *IFN-γ* Interferon-gamma, *Trp* Tryptophan, *Asn* Asparagine, *Glu* Glutamate, *T*_*reg*_* cell* Regulatory T cell, *mGlutR1* Metabotropic glutamate receptor 1, *Leu* Leucine, *LARS2* Leucine-tRNA-synthase-2

### T cell

Proverbially, naïve CD8^+^ T cells differentiate into memory CD8^+^ T cells through the stages of initial activation, expansion, and sorting of the immune response, a process that is tightly regulated by cell-surface receptors, soluble factors, and transcriptional programs and associated with metabolic reprogramming [168]. As proof, signaling roles for the metabolism of lipid-derived molecules such as prostaglandins in T cell responses have been reported [169]. Scavenger receptor CD36 uptakes lipids and promotes T_reg_ cell function, but inhibits the killing effect of CD8^+^ T cells in the TME [161, 170]. Interestingly, CD28 co-stimulatory signaling upregulates mammalian target of rapamycin (mTOR) complex 1 (mTORC1), which is activated in a T cell receptor (TCR)-dependent manner, suggesting that the CD28 molecule impacts lipid anabolism and immune responses of effector T cells [171, 172]. The involvement of mTORC1 in the downregulation of CXCR4 and inhibition of bone marrow infiltration of CAR-T cells and elimination of acute myeloid leukemia (AML) may provide a potential reason for the limited efficacy of cellular therapies in AML [173]. However, Werter et al. observed in patients with metastatic renal cell carcinoma (RCC) treated with everolimus that cyclophosphamide attenuates mTOR-mediated regulatory T-cell expansion without affecting clinical outcomes [174]. Likewise, Braun et al. reported that no immune infiltration phenotype was observed to correlate with clinical benefit in 66 patients with advanced RCC (clear cell histology, mTOR inhibition group) [175]. IL-15 signaling drives upregulation of CPT1a expression to promote FAO, whereas IL-7 signaling induces glycerol uptake to promote triacylglycerol synthesis and FAO, thus supporting the longevity of memory CD8^+^ T cells [167, 176]. Short-chain fatty acids (SCFAs) reconfigure metabolism to allow activated T cells to take up and oxidize more FAs, thereby transforming them into memory CD8^+^ T cells with long-term viability [177]. Additionally, cholesterol depletion promotes the generation of IL-9-producing CD8^+^ T cells (Tc9 cells, potent anti-tumor immune inducers) by modulating the activity of the transcription factor liver X receptor (LXR) [163]. Mediated by a metabolite (oxysterol 7α,25-dihydroxycholesterol), EBI2 enhances T follicular helper cell fate by promoting interaction with IL-2-quenched dendritic cells [178]. The LCA derivative 3-oxoLCA inhibits TH17 cell differentiation by binding to the TH17 cell-specific transcription factor RORγt (retinoic acid receptor-associated orphan receptor γt) [179]. In an obesity-associated breast tumor model, STAT3 activation induces FAO in CD8^+^ T cells and impairs CD8^+^ T cell effector function [81]. In addition, stimulated TCRs activate PI3K-Akt and the mTOR signaling pathway, which subsequently induces FA and mevalonate synthesis, whereas posttranslational modifications dependent on mevalonate metabolism are essential for T_reg_ cell activation and the establishment of immune tolerance [180, 181]. In short, these studies adequately portray the flexible metabolic plasticity and subpopulation plasticity of T cells.

### B cell

B cells are important components of adaptive immunity and the relationship between fate determination of B cells and glucose or glutamine metabolic pathways has received much attention [14, 182]. Metabolic reprogramming of activated B cells has been reported to require the involvement of the sterol regulatory binding protein (SREBP) pathway [183, 184]. CD37 inhibits the FA transporter FATP1 through molecular interactions, which subsequently leads to the inhibition of FA metabolism in aggressive B cell lymphomas [162]. In addition, the findings of Cheng et al. linked cellular metabolism to B cell antigen receptor signaling reveal that fumaric acid inhibits B-cell activation and function by directly inactivating the tyrosine kinase LYN [185]. Naïve B cells treated with 25-hydroxycholesterol inhibit IL-2-mediated B cell proliferation, leading to a significant reduction in IgA [164]. 24-hydroxycholesterol is involved in angiogenesis and in the development of pancreatic neuroendocrine tumors [186]. Oxysterol gradient, produced in lymphoid stromal cells, binds to the upregulated EBI2 receptor on the surface of B cells and promotes the movement of B cells in and out of follicles in response to antigenic stimulation [187]. Sphingosine 1-phosphate (S1P) is a metabolic intermediate of sphingomyelin that functions as a multi-effector lipid mediator in tissues such as the circulatory, nervous, and lymphatic systems [188, 189]. S1P/S1P1 signaling has been reported to help guide the release of nascent immature B cells from the bone marrow into the bloodstream [190, 191]. Significant reductions in germinal center responses, antibody production, mitochondrial mobilization, and OXPHOS have been demonstrated in CD36-deficient B cells [192]. Therefore, understanding B cell metabolic patterns is expected to provide therapeutic targets for B cell-associated immune processes.

### Macrophage

In vitro studies performed in the context of pro- or anti-inflammatory activation highlighted the metabolic plasticity of macrophages [15]. Evidence suggests that M1 macrophages prefer to receive activation signals *via* glycolysis, whereas M2 macrophages favor mitochondrial metabolism and FAO [127, 193]. CD36 molecules, which act as signaling receptors and FA transporters, are known to regulate the metabolism and fate of immune cells, particularly macrophages and T cells [194]. In addition, macrophage Acly deficiency stabilizes atherosclerotic plaques [195]. Previously reported Tissue-resident macrophages (TRMs) are diverse cell families, which generally present long-lived and self-renewing [196, 197]. TRMs are exposed to and adapt to many tissue-specific growth factors and actively participate in cellular metabolism to maintain tissues and organism balance [198, 199]. For example, adipose tissue TRMs contribute to metabolic processes such as insulin sensitivity, adipogenesis, and adaptive thermogenesis [200]. Lack of peroxisome proliferator-activated receptor-γ (PPARγ) targeting or disturbed lipid metabolism in alveolar macrophages leads to pathologic accumulation of surface-active substances in the lungs [201]. Similarly, lowering systemic cholesterol levels with PPARγ agonists, lung X receptor agonists, or statins reduces the pathologic changes of proteolytic disease in mice [202]. Hence, the above reports confirm the importance of variation in metabolism-related targets for functional alterations in macrophages, as well as macrophage regulation of metabolic processes.

### Dendritic cell

Differentiation of human monocytes to dendritic cells (DCs) is accompanied by increased expression of PPARγ, a key transcription factor controlling lipid metabolism [203, 204]. The deoxycholic acid derivative 3β-hydroxydeoxycholic acid (isoDCA) inhibits NR1H4 transcriptional activity in DCs and subsequently induces Foxp3 expression [16]. MYC is a transcription factor that promotes the expression of genes encoding proteins in the glycolytic pathway [205]. However, MYC expression is downregulated during DC development with the emergence of MYCL expression in conventional DCs (cDCs) progenitor cells [206]. Resting GM-CSF-induced bone marrow-derived DCs (BMDCs) differ from activated DCs in their weaker ability to interact with and activate T cells. BMDCs have been shown to use FAO to promote OXPHOS [207]. Nevertheless, it is elusive whether resting cDCs or plasmacytoid dendritic cells (pDCs) similarly fuel OXPHOS *via* FAO. Speaking generally, further explorations regarding the network of interactions between DCs and metabolism allow researchers to achieve a comprehensive understanding of immune metabolism in cancer.

### Natural killer cell

The process by which natural killer (NK) cells achieve functional maturation and self-tolerance is known as NK cell education (also known as NK cell licensing), and changes in cellular metabolism are associated with this NK cell education process [208]. Resting mouse NK cells have been shown to have a low basal metabolic rate, maintaining low levels of glycolysis and OXPHOS [209, 210]. Notably, prolonged exposure of human NK cells to IL-15 in vitro results in a reduced metabolic rate [211]. In human NK cells, inhibition of amino acid uptake by SLC1A5 and SLC7A5 prevents IFNγ production as well as degranulation after cross-linking of the activating receptor NKG2D17 antibody [212]. CD36 researchers investigated changes in NK cell function in hyperlipidemic mice, which found that DCs with more lipids in the cytoplasm relied on ROS to increase the expression of PD-L1, TGF-β1, and NKG2D ligands and inhibit NK cell activity [213]. Pre-NK cells (CD11b^low^CD27^hi^) undergo a proliferative burst that is associated with the expression of the amino acid transporter SLC3A2 and transferrin receptor [209, 214]. Indeed, NK cells do not use glutamine as a fuel to drive OXPHOS, and inhibition of glutaminase does not inhibit OXPHOS or affect the function of NK cell effectors [210, 215]. Furthermore, whether NK cells use FAs as a fuel source has not been extensively studied. Interestingly, the accumulation of excess FAs in NK cells is thought to be detrimental to NK cell metabolism and function [216]. Not to be overlooked, it is generally accepted that NK cells have long-term functions and are characterized by immune memory [217–219]. An important process in the formation and self-renewal of memory NK cells is the restoration of mitochondrial metabolic function (achieved by removing damaged mitochondria through mitochondrial autophagy) [220, 221]. Furthermore, CD16 cross-linking on adaptive NK cells induces stronger mTORC1 activity compared to non-adaptive NK cells [222]. Overall, normal cellular metabolic drives are critical for NK cell development (including NK cell education) and immune function, but research in this area may lead to non-negligible therapeutic opportunities.

### Neutrophil

As the most common cell type among leukocytes, neutrophils are considered to be the most abundant innate immune effector cells in the human immune system [160, 223, 224]. Tumor-associated neutrophils (TANs) have become an important part of the tumor microenvironment and play a double-edged role [165, 225, 226]. Under basal conditions, neutrophils predominantly undergo glycolysis with little mitochondria and oxygen exposure deleteriously affects neutrophil viability [227–229]. Defects in neutrophil glucose cycling (e.g., G6P transporter deletion) result in reduced glucose uptake and lower intracellular G6P, and also impair energy metabolism [230–232]. Arginase 1 (ARG1) blockade in combination with immune checkpoint inhibitors promotes CD8^+^ T cells in pancreatic ductal adenocarcinoma (PDAC) in vitro [166]. The induction of ER stress in neutrophils upregulates the expression of LOX1, a scavenger receptor involved in lipid metabolism, as well as potent immunosuppressive activity [233]. Neutrophil supply is tightly regulated by three mechanisms: phagocytosis, degranulation, and release of neutrophil extracellular traps (NETs) [160]. Mitochondrial ROS oxidize NET DNA, thereby enhancing its ability to activate the stimulator of interferon genes (STING) signaling and drive IFN production by pDCs [234, 235]. Based on the diversity and plasticity of neutrophil metabolism, it is reasonable to hypothesize that targeting TANs and NETs may become an integral and important component of immunotherapy.

### Metabolically driven immunogenic cell death

Cancer immunoediting is the process by which immune cells constrain and promote tumor development through three phases: elimination, homeostasis, and escape [236, 237]. Through these processes, cancer immunogenicity declines as a result of the synergistic action of primary and adaptive immunosuppressive mechanisms. Immunogenic cell death (ICD) is a type of regulatory cell death that is sufficient to activate adaptive immunity in an immunocompetent host [238, 239]. Upon induction of ICD, dying tumor cells release or expose damage-associated molecular patterns (DAMPs). Immunogenic chemotherapy and radiotherapy both upregulate the expression of MHC class I and class II molecules on the surface of tumor cells, thereby enhancing their antigenicity [240, 241]. Of note, increasing numbers of ICD inducers have positively interacted with ICIs or other immunotherapies in cancer patients [242, 243]. Zhou et al. reported that ICD induction enhances anti-tumor immunity and inhibits tumor immune evasion through CD47 blockade, which may be expected to improve cancer chemoimmunotherapy [244]. Based on the ability to trigger cancer cell death and danger perception, ICD inducers can be categorized into two types, including type I (generating reactive oxygen species) and type II (inducing endoplasmic reticulum stress) inducers [245, 246]. Doxorubicin-induced ICD is caspase-dependent, and both doxorubicin and mitoxantrone induce tumor cells to expose CRT, secrete ATP, and release HMGB1 [247–250]. From the perspective of cancer immunotherapy, the exploration of the characterization of metabolism-associated ICD, the underlying cell biology, and the pathways by which immune effector cells sense ICD will be one of the important plates in future clinical strategies.

### Immunoediting-driven metabolic adaptations in cancers

#### Cancer-immunity cycle and cancer-immunometabolism subcycle

**Fig. 4:**
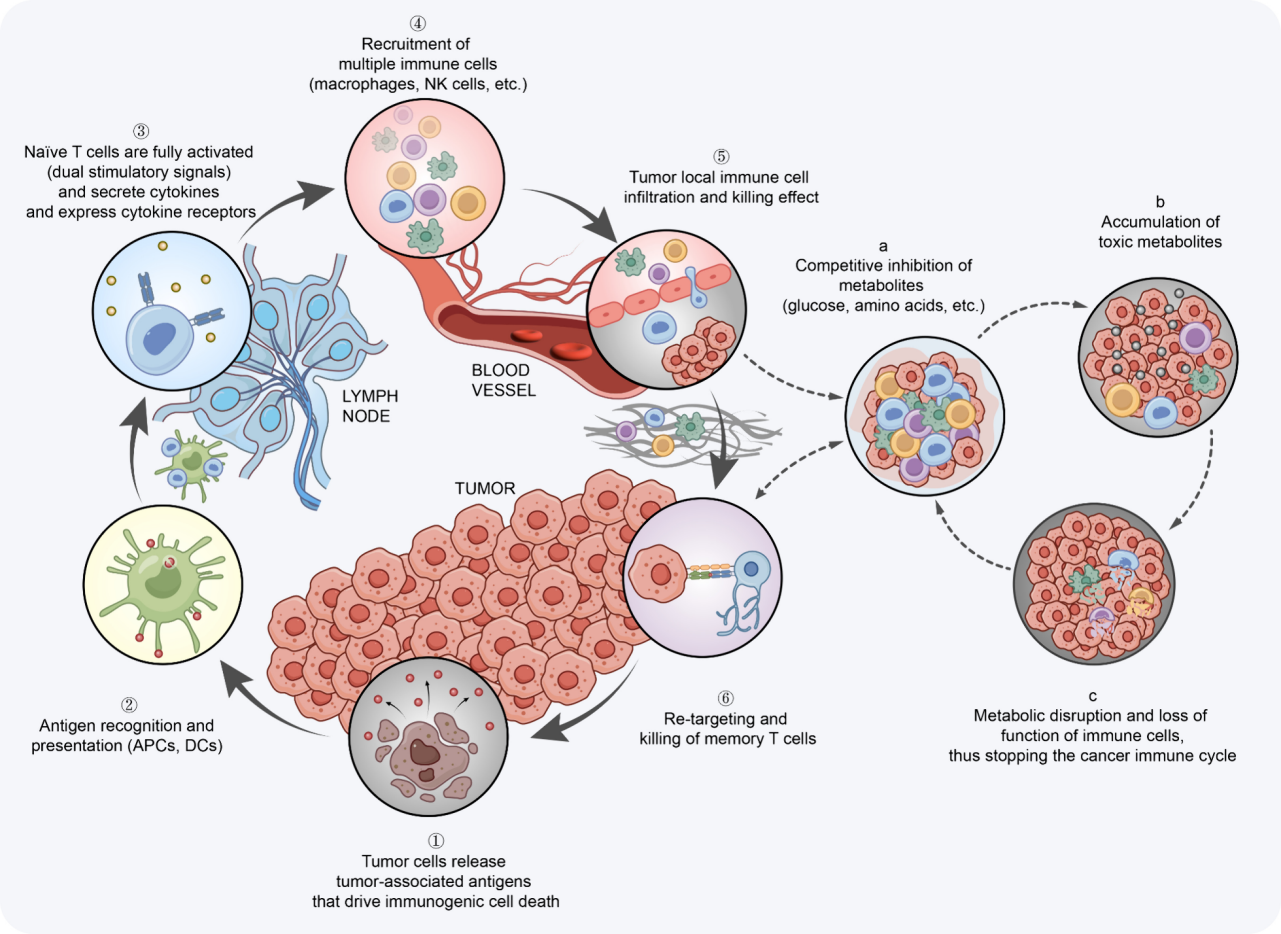
Disruptor of the virtuous cycle: the cancer-immunometabolism subcycle. In fact, antigen release occurs consistently in most patients with malignant tumors. Nevertheless, the CI cycle of Chen et al. suggests that it does not imply that the inevitable occurrence of cancer cell death events (the left cycle). Metabolic-related factors may be responsible for the disruption of the CI cycle. Considering the cancer-immunometabolism subcycle (the right cycle) proposed in this review, it is reasonable to assume that metabolism-related factors may contribute to the interruption of the CI cycle (the left cycle). When immune cells reach the tumor microenvironment through the vascular endothelium or basement membrane, nutrient deprivation as well as accumulation of local toxic substances accelerate the formation of tumor immunosuppressive microenvironment. Thereby, the infiltrating immune cells become dysfunctional, such as altered macrophage polarization, diminished killing effect of T cells and NK cells, and formation of NETs, and so on. As the result, the CI cycle is impaired and failed to stimulate a potent and sustainable immune response. CI cycle, cancer-immunity cycle; NK cells, natural killer cells; NETs, neutrophil extracellular traps

Effective anti-tumor immune responses must initiate a series of step events and cycle back and forth, which are termed “cancer-immunity cycle” (CI cycle) [251, 252]. The left circle of Fig. 4 delineates the warrant steps involved in the CI cycle. Briefly, tumor antigens are presented by DCs and recognized by effector T cells to kill the tumor cells, and the killed tumor cells release more tumor-associated antigens to further promote the breadth and depth of the immune response [252]. In this cycle, the balance of the ratio of T effector cells to T_reg_ cells is critical to the outcome. However, in cancer patients, the CI cycle often does not work optimally [252]. Namely, tumor antigens may not be detected or effectively activate DCs, DCs may recognize antigens as self-antigens and subsequently escape immune surveillance, and T cells may not properly home to the tumor bed for killing [253].

Nonetheless, metabolic disorders in cancer cells further create a vicious circle by creating a microenvironment that contains tumor metabolites conducive to cancer cell growth [254]. Scientists from Switzerland and other institutions have found that immune cell surveillance of cancer may itself induce metabolic adaptations in early-stage tumor cells, while also promoting their growth and giving them the ability to suppress the body’s lethal immune response [255]. In a workflow of influences on CD8^+^ T cell differentiation in cancer, Giles et al. argued that metabolism should be incorporated as a fourth signal to better execute the CI cycle [256]. In light of this, we propose the scenario of the cancer-immunometabolism subcycle (Fig. 4, the right circle). Filtrating the fundamental features of the CI cycle will help us to accurately characterize our understanding of the cancer immune response, and these insights will have profound implications for the establishment and application of immunometabolism.

### Immunotherapy and precision medicine drug development

In oncology, immunotherapy and precision medicine drug development are in the ascendant. In this light, only about one-third of patients respond to immunotherapy, with the type of immunity playing a decisive role [251]. Table 2 briefly list the clinical prospects explored under several metabolic studies. VEGF blockade combined with tumor-derived glutamate has been found to induce systemic and intra-tumoral immunosuppression, and this effect can be prevented by T_reg_ depletion, thereby enhancing anti-tumor efficacy [9]. Of interest, JHU083 was able to synergize with immunotherapy to enhance infiltration, proliferation, activation, and function of effector T cells in tumors [52]. Reducing GCPII expression through genetic alterations or pharmacological inhibition of glutamate carboxypeptidase II (GCPII) leads to reductions in glutamate concentration and tumor growth, which are enhanced by targeting GCPII in combination with glutaminase inhibition [257]. The combination of the drug with the lipoprotein transport pathway allows optimized lipophilic parent drug to be transported via the lymph, reducing ineffective exposure to the drug and subsequently enhancing efficacy [82, 258]. In addition, it may also target specific disease reservoirs in lymphatic vessels, providing advantages for advanced immunotherapeutic cancer strategies [259].

**Table 2:** Metabolic perspectives in immunotherapy and drug development

**Candidate agents/inhibitors**	**Targets**	**Monotherapy or in combination**	**Mechanisms**	**Refer to Refs.**
JHU083	Glu	Monotherapy	Alleviating tumor growth in various solid tumor models and significantly increased mouse survival	[52]
C75	FASN	In combination with the PI3Kα-specific inhibitor CYH33	Enhanced M1 polarization of macrophages and activity of CD8+ T cells that synergistically inhibit tumor growth and enhance host immunity	[124]
L-Arginine	BAZ1B, PSIP1, and TSN	Monotherapy	Enhances T-cell survival and anti-tumor activity	[47]
Anti-hARG1 mAbs	ARG1	In combination with ICIs	ARG1 blockade in combination with ICIs has a therapeutic effect by increasing tumor infiltration of activated CD3^+^ T cells in vitro	[166]
V-9302	glutamine transporter	Monotherapy	Inhibiting the update of glutamine in TNBC cells and but does not affect anti-tumor T cells	[53]
VISTA	PSGL-1	In combination with anti-PD-1	Alteration of acidic pH due to accumulation of local glycolytic products in tumors, reverses T cell suppression, and triggers immune rejection	[101]
Citrate	PFK1 and PFK2	In combination with Cisplatin	Reducing ATP production, induces apoptosis, and sensitizes cells to cisplatin treatment	[86]
BPTES	GCPII	Monotherapy or in combination with glutaminase	Decreasing glutamate concentration and inhibits tumor growth	[257]
Rapamycin	mTORC1	Monotherapy	Facilitating EpCAM CAR-T cell bone marrow migration by upregulating CXCR4	[173]
			Impairing DC differentiation and survival	[260]
			Specifically interferes with GM-CSF signaling in human DCs	[261]
2DG	HK2	Monotherapy	Inhibition of IFN-γ production and granzyme B expression by NK cells	[262, 263]
Avasimibe	ACAT-1	Monotherapy	Up-regulation of cholesterol biosynthetic enzymes increases plasma membrane cholesterol levels and improves synaptic function in CD8^+^ T cells	[70]

*PI3Kα* Phosphatidylinositol 3-kinase alpha, *hARG1* Human arginase 1, *ARG1* Arginase 1, *ICIs* Immune checkpoint inhibitors, *EpCAM* Epithelial cell adhesion molecule, *BPTES* Bis-2-(5-phenylacetamido-1,3,4-thiadiazol-2-yl) ethyl sulfide, *Glu* Glutamate, *GM-CSF* Granulocyte-macrophage colony-stimulating factor, *2DG* 2-deoxyglucose, *IFN-γ* Interferon-gamma, *VISTA* V-domain immunoglobulin suppressor of T cell activation, *TNBC* Triple-negative breast cancer, *ACAT-1* Acetyl-CoA acetyltransferase-1

Given the Leu nutritional preference exhibited by leucine-tRNA-synthase-2-expressing B (LARS B) cells, Wang et al. proposed a leucine dieting regimen, which is considered to be a favorable option for colorectal cancer treatment [44]. In an allogeneic hematopoietic cell transplantation model, ceramide synthase 6-deficient T-cell proliferation and IFN-γ production were blocked, suggesting that targeting ceramide synthesis is expected to improve allogeneic hematopoietic cell transplantation therapy [264]. Additionally, the differentiation and survival of human monocyte-derived DCs were impaired by rapamycin [260, 261]. Metabolic inhibitor 2-deoxyglucose (2DG) limits glycolysis and OXPHOS and inhibits IFN-γ production and granzyme B expression in mouse and human NK cells [262, 263]. Neutrophil extracellular traps accumulate in the peripheral vasculature of tumor-bearing animals and impair organ function, and treatment with the autophagy-based inhibitor chloroquine blocks peripheral infiltration of neutrophils [265]. Currently, while next-generation checkpoint inhibitors may provide some benefit, it seems unlikely that they alone will overcome the hurdles specific to the CI cycle and immunotherapy. On the road to exploring immunotherapy, “metabolic checkpoints” also require more attention.

### Pending challenges and clinical concerns

Based on the Warburg effect being observed in a variety of solid tumors, oncologists have been trying to reduce glucose utilization in tumors as a treatment for decades [110]. However, relevant attempts have not yet reached the stage of clinical application, such as attempts with hexokinase inhibitors. However, immunotherapy for tumors is on the rise. Herein, we present the following topics along with clinical issues that we hope will overcome in the future. (i) In the crosstalk of tumor metabolism and immune regulation, how do some key pathways, such as ROS signaling, TCR signaling, and co-stimulatory signaling, regulate each other, what are the important targets, are the differences between individuals significant, and are there clinical transformations (such as nutritional interventions) accessible? (ii) in fact, some carcinogenic signaling pathways, such as HIF-1α and RAS/PI3K/AKT, may generate drug resistance by enhancing aerobic glycolysis in cancer cells (the “Warburg effect”) [86]. This metabolic pattern that promotes cancer cell development and invasiveness also induces enhanced extracellular acidity, whereas most drugs are weakly basic molecules. On the one hand, citrate could act as a physiological inhibitor of PFK1 and PFK2, inhibiting glycolysis; On the other hand, citrate leads to acid-base imbalance in the extracellular environment, weakening the effective infiltration concentration of alkaline drug molecules in the local tumor microenvironment, and even triggering multi-drug resistance events. For example, sodium bicarbonate has been observed to increase responsiveness to immunotherapy in models of melanoma and pancreatic cancer [266]. Therefore, the effect of the disturbance of acid-base balance in the local microenvironment caused by metabolism on immunotherapy is still worth exploring. (iii) In a series of enzymatic reactions of immune metabolism, are there key enzymatic molecules that finely and efficiently regulate immune surveillance and killing effects, produce a dominant effect on the tumorigenesis and progression (exerting a similar role as “rate-limiting enzymes”)? (iv) How to determine the difference between metabolic studies based on tumor animal models (such as mice) and patients’ tumor microenvironment metabolism? (v) Metabolic enzymes maintain the metabolic balance of the whole body in a normal body. For instance, inhibitors of FASN have shown severe systemic side effects such as weight loss and anorexia in some clinical studies [267]. Therefore, how can strategies be developed to target dysregulated metabolism in cancer through nutritional interventions, and thus improve anti-tumor immunity? How far is a personalized treatment strategy for tumor metabolic inhibitors from clinical patients? (vi) Metabolic changes caused by aging have brought about a series of changes in pathophysiological processes, and the immune state of the body is inevitably affected. Which metabolic changes ultimately lead to an increased risk of cancer during this process? Can this condition be avoided or slowed down with age? (vii) Long-term accumulation of abnormal metabolism or sudden but intense metabolic changes, which has a greater impact on tumors, the former or the latter?

### Concluding remarks

Undoubtedly, metabolic reprogramming is not unique to tumor cells, and immune cells share this feature. Immunology and oncology investigators are increasingly aware that different stages of immune cell activation coincide with different types of cellular metabolism. We hope to initiate or restart anti-tumor immune responses and maintain self-circulation while ensuring that unrestrained autoimmune inflammation is avoided. Herein, we have learned that there are checkpoints and inhibitors at every step of metabolism and immunity that impede the anti-tumor response from proceeding and expanding further, and that effective approaches are selective processing of patients at different limiting steps to adapt to tumor evolution. Immunophenotype variability and plasticity have profoundly inspired researchers to explore new directions for cancer treatment. Likewise, as is currently understood in the era of tumor immunology, it is reasonable to assume that metabolism is far less accurately and comprehensively delineated than it is perceived to be. The concept of immunometabolism could assist in the development of efficient treatment options, as well as the understanding of immune regulatory mechanisms focusing on metabolic perspectives will bring out a profound impact on the design of clinical therapies.

## Data Availability

No datasets were generated or analysed during the current study.

## Abbreviations

ATP: Adenosine 5’-triphosphate (ATP)
ROS: Reactive oxygen species
Leu: Leucine
Arg: Arginine
TME: Tumor microenvironment
Glu: Glutamate
Gln: Glutamine
CNS: Central nervous system
Trp: Tryptophan
Asn: Asparagine
IDO: Indoleamine 2,3-dioxygenase
Kyn: Kynurenine
IFN-γ: Interferon-gamma
MCs: Mesenchymal cells
NK: Natural killer
ER: Endoplasmic reticulum
ACC1: Acetyl-CoA carboxylase 1
AMPK: AMP-activated protein kinase
CSCs: Cancer stem cells
αKG: α-ketoglutarate
cDC1: Conventional type 1 dendritic cell
HK2: Hexokinase 2
GC: Gastric cancer
PD-L1: Programmed cell death ligand 1
AKAP: A-kinase anchoring protein
EMT: Epithelial-mesenchymal transformation
PFK1: Phosphofructokinase 1
F6P: Fructose-6 phosphate
F1,6BP: Fructose-1, 6-diphosphate
PFKL: Phosphofructokinase-1 liver type
TGF-β: Transforming growth factor-β
FA: Fatty acid
FASN: Fatty acid synthase
FAO: Fatty acid oxidation
PI3Kα: Phosphatidylinositol 3-kinase alpha
LAL: Lysosomal acid lipase
MDSCs: Myeloid-derived suppressor cells
PC: Pyruvate carboxylase
Acly: ATP citrate lyase
IDH: Isocitrate dehydrogenase
d-2HG: d-2-hydroxyglutarate
HAase: Hyaluronidase
HA: Hyaluronic acid
Mtor: Mammalian target of rapamycin
mTORC1: mammalian target of rapamycin complex 1
TCR: T cell receptor
AML: Acute myeloid leukemia
RCC: Renal cell carcinoma
SCFAs: Short-chain fatty acids
LXR: Liver X receptor
SREBP: Sterol regulatory binding protein
T_reg_ cell: T regulatory cell
S1P: Sphingosine 1-phosphate
TRMs: Tissue-resident macrophages
DCs: Dendritic cells
PPARγ: Peroxisome proliferator-activated receptor-γ
cDCs: conventional DCs
pDCs: plasmacytoid dendritic cells
NSCLC: Non-small cell lung cancer
BMDCs: Bone marrow-derived DCs
isoDCA: 3β-hydroxydeoxycholic acid
TANs: Tumor-associated neutrophils
NETs: Neutrophil extracellular traps
ARG1: Arginase 1
PDAC: Pancreatic ductal adenocarcinoma
pDCs: plasmacytoid dendritic cells
STING: Stimulator of interferon genes
ICD: Immunogenic cell death
DAMPs: Damage-associated molecular patterns
CI cycle: Cancer-immunity cycle
GCPII: Glutamate carboxypeptidase II
LARS B cells: Leucine-tRNA-synthase-2-expressing B cells
2DG: 2-deoxyglucose
